# A novel variant in *COX16* causes cytochrome c oxidase deficiency, severe fatal neonatal lactic acidosis, encephalopathy, cardiomyopathy, and liver dysfunction

**DOI:** 10.1002/humu.24137

**Published:** 2020-11-30

**Authors:** Liesbeth T. M. Wintjes, Maina Kava, Frans A. van den Brandt, Mariël A. M. van den Brand, Oksana Lapina, Yngve T. Bliksrud, Mari A. Kulseth, Silja S. Amundsen, Terje R. Selberg, Marion Ybema‐Antoine, Omar A. Z. Tutakhel, Lawrence Greed, David R. Thorburn, Trine Tangeraas, Shanti Balasubramaniam, Richard J. T. Rodenburg

**Affiliations:** ^1^ Department of Laboratory Medicine, Translational Metabolic Laboratory Radboud Centre for Mitochondrial Medicine, Radboudumc Nijmegen The Netherlands; ^2^ Department of Neurology Perth Children's Hospital Perth Western Australia Australia; ^3^ School of Pediatrics and Child Health University of Western Australia Perth Western Australia Australia; ^4^ Department of Pediatrics Radboud Centre for Mitochondrial Medicine Radboudumc Nijmegen The Netherlands; ^5^ Department for Radiology and Nuclear Medicine Oslo University Hospital Oslo Norway; ^6^ Norwegian National Unit for Diagnostics of Congenital Metabolic Disorders, Department of Medical Biochemistry Oslo University Hospital Oslo Norway; ^7^ Department of Medical Genetics Oslo University Hospital Oslo Norway; ^8^ Department of Pediatrics Ostfold Hospital Trust Kalnes Norway; ^9^ Department of Clinical Biochemistry PathWest Perth Western Australia Australia; ^10^ Murdoch Children's Research Institute and Victorian Clinical Genetics Services Royal Children's Hospital Melbourne Victoria Australia; ^11^ Department of Pediatrics University of Melbourne Melbourne Victoria Australia; ^12^ Norwegian National Unit for Newborn Screening, Division of Pediatric and Adolescent Medicine Oslo University Hospital Oslo Norway; ^13^ Western Sydney Genetics Program The Children's Hospital at Westmead Sydney New South Wales Australia

**Keywords:** assembly factor, cardio‐encephalopathy, COX16, mitochondrial complex IV deficiency, OXPHOS

## Abstract

COX16 is involved in the biogenesis of cytochrome‐c‐oxidase (complex IV), the terminal complex of the mitochondrial respiratory chain. We present the first report of two unrelated patients with the homozygous nonsense variant c.244C>T(p. Arg82*) in *COX16* with hypertrophic cardiomyopathy, encephalopathy and severe fatal lactic acidosis, and isolated complex IV deficiency. The absence of COX16 protein expression leads to a complete loss of the holo‐complex IV, as detected by Western blot in patient fibroblasts. Lentiviral transduction of patient fibroblasts with wild‐type *COX16* complementary DNA rescued complex IV biosynthesis. We hypothesize that COX16 could play a role in the copper delivery route of the COX2 module as part of the complex IV assembly. Our data provide clear evidence for the pathogenicity of the *COX16* variant as a cause for the observed clinical features and the isolated complex IV deficiency in these two patients and that COX16 deficiency is a cause for mitochondrial disease.

## Graphical abstract

We present the first report of two unrelated patients with the homozygous nonsense variant c.244C>T(p. Arg82*) in COX16 with hypertrophic cardiomyopathy, encephalopathy and severe fatal lactic acidosis, and isolated complex IV deficiency. Our data provide clear evidence for the pathogenicity of the COX16 variant as a cause for the observed clinical features and the isolated complex IV deficiency in these two patients and that COX16 deficiency is a cause for mitochondrial disease.

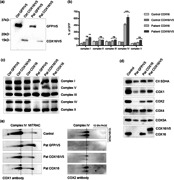

## 1 BRIEF REPORT

Cytochrome c oxidase (complex IV; COX; EC 1.9.3.1) is the terminal complex of the mitochondrial respiratory chain (RC). It catalyzes the electron transfer from cytochrome c to molecular oxygen, coupled to proton translocation across the mitochondrial inner membrane. This generates the electrochemical gradient that drives ATP production by ATP synthase. Complex IV is embedded in the inner mitochondrial membrane. It is a multimeric complex, composed of 14 subunits, 3 catalytic core subunits COX1, COX2, and COX3 which are encoded by the mitochondrial genome, and 11 subunits by the nuclear genome (Balsa et al., 2012; Pitceathly & Taanman, 2018). The assembly of complex IV is a finely tuned process, which occurs in a modular manner (Timon‐Gomez et al., 2018; Vidoni et al., 2017). The COX catalytic core subunits form relatively independent preassembly modules, each with their subunit‐specific assembly factors. These modules are joined to form the holo‐complex IV. The role of the various assembly factors in this assembly process is still not fully understood. More than 30 different assembly factors have been described to date (Signes & Fernandez‐Vizarra, 2018; Timon‐Gomez et al., 2018), and the number of potential genes encoding for assembly factors is still growing (Timon‐Gomez et al., 2018). Pathogenic variants in the genes encoding the complex IV subunits and assembly factors lead to a complex IV deficiency, the second most abundant isolated RC deficiency causing 20%–25% of all mitochondrial disorders (Debray et al., 2007; Scaglia et al., 2004). In many cases, it results in severe, often fatal, early‐onset neuromuscular disorders. The majority of the deficiencies are caused by variants in nuclear DNA genes encoding proteins involved in the biogenesis of complex IV (Ghezzi & Zeviani, 2018; Shoubridge, 2001).

The first indications that COX16 may act as an assembly factor for complex IV came from research performed in *Saccharomyces cerevisiae* (Carlson et al., 2003; Su & Tzagoloff, 2017). In recent research using human embryonic kidney 293 (HEK293) cells it was shown that COX16 participates in the complex IV biogenesis (Aich et al., 2018; Cerqua et al., 2018). However, its precise role in complex IV assembly is still not completely clear.

In this report, we describe two unrelated patients with lactic acidosis, hypertrophic cardiomyopathy, and a complex IV deficiency in which a novel pathogenic homozygous nonsense variant was detected in *COX16* by whole‐exome sequencing (WES).

The first patient (Subject 1) was a full‐term male infant (G7P7) of a healthy non‐consanguineous Caucasian couple. He had symmetrical intrauterine growth retardation with a birth weight (BW) of 1700 g, required resuscitation with oxygen, and intermittent positive pressure ventilation. Severe lactic acidosis was noted at birth with a cord pH of 7.06, lactate 20 mmol/L, and base excess −20 mmol/L which persisted despite bicarbonate infusion and THAM. Initial severe hypoglycemia with plasma glucose less than 1.1 mmol/L was corrected. The infant was encephalopathic, extremely floppy, and areflexic with no clinical seizures. He had multisystemic involvement with liver failure and coagulopathy, moderately dilated and hypertrophic right ventricle, and bilateral congenital sensorineural hearing loss. There were no cataracts, optic atrophy, or retinitis pigmentosa observed in the patient. The lactic acidosis remained fixed and persistent at 25–35 mmol/L. The baby remained obtunded and ventilator dependent and succumbed shortly aftercare was withdrawn at 55 h. Blood for DNA was collected on Day 2, and muscle and skin were collected on Day 3 of life. Magnetic resonance imaging (MRI) of the brain showed multiple areas of supratentorial and brainstem infarcts with generalized cerebral edema (Figure S1a,b). Lactate peak was observed on magnetic resonance spectroscopy (Figure S1c). EEG showed an abnormal background. Specific and repeated metabolic investigations revealed a blood lactate 35.7 mmol/L (NR < 2.1), pyruvate 0.33 mmol/L (NR < 0.12), and L/P ratio 109 (NR 14–28). Plasma amino acids showed significant increases in glutamine (1700 μmol/L), alanine (1500 μmol/L), and proline (1400 μmol/L); methionine and tyrosine were also moderately increased. Plasma ammonium (37 μmol/L) was normal. Urine organic acids analysis showed markedly increased lactate, with a fixed moderate ketonuria, and increased 4‐hydroxyphenyllactate; the urine polyols showed secondary increases in all fractions; other tests were normal. The plasma acylcarnitines showed borderline increases (in *C4, C6, C8, C10, C16*, and *C18*) in a nonspecific pattern; Very long chain fatty acid, phytanate, transferrin, and apoC‐Ill isoforms were normal. Serum creatine kinase was 3000 U/L (NR < 450), serum iron studies were normal. Liver function tests showed 2–3‐fold increases in the transaminases, renal functions were mildly abnormal and plasma cortisol, insulin, and growth hormone responses to the hypoglycemic were appropriate.

The second patient (Subject 2) was a full‐term (Week 40 + 3) female infant (G2P2) of a healthy non‐consanguineous Caucasian couple. BW 3210 g, head circumference (HC) 35 cm, length 48 cm, Apgar score 9–10–10. After 24 h of life, she was transferred to the neonatal intensive care unit due to tachypnoea, irritability with a high‐pitched cry, regurgitations, and hypoglycemia (1 mmol/L). The hypoglycemia was quickly resolved by glucose infusion. Severe lactic acidosis (pH 7.13, pCO_2_: 3.1 kPa, HCO_3_^−^: 7.7 mmol/L, and base excess: −21.5 mmol/L, lactate: 16 mmol/L) was corrected by bicarbonate infusion. At 40 h of age she was clinically unremarkable except slightly hypotonic; no dysmorphic signs were noted. Blood samples showed pH 7.38, pCO_2_: 3.3 kPa, HCO_3_^−^: 15 mmol/L, lactate: 7.6 mmol/L, CK: 1492 U/L, ALAT: 46 U/L, ASAT: 125 U/L, albumin: 32 g/L, INR: 2.0–1.1 (*N*‐ratio), plasma ammonium 68–22 μmol/L. Echocardiography and brain ultrasonography were normal. Organic acids in urine showed a “mitochondrial profile” with elevated excretion of lactate, succinic acid, fumaric acid, ethylmalonic acid, 2‐keto‐glutaric acid, dicarboxylic acids, 3‐OH‐dicarboxylic acids. In addition, the excretion of *p*‐OH‐phenyllactate and possibly vanillactic acid were increased. Plasma long‐chain acylcarnitines showed elevations of which C12‐carnitine (0.38 µmol/L [0.030–0.16]) and C14‐carnitine (0.36 µmol/L [NR: 0.020–0.12]) were most pronounced, C10‐OH‐, C10‐, C8‐, C6‐, C5DC‐, C4‐OH‐, C3DC‐ and C2‐carnitine were slightly raised. Amino acids in plasma showed an elevated concentration of proline 467 µmol/L (NR 101–272) and normal alanine 354 µmol/L (NR 120–520). Amino acids in cerebrospinal fluid (sampled at 58‐h age) showed elevation of several amino acids including glutamine 778 μmol/L (NR 374–777), proline 2 μmol/L (NR < 1.1), and alanine 53 μmol/L (NR 18–40). Spinal‐lactate was 3.3 mmol/L (NR 0.8–2.8), pyruvate 0.17 mmol/L (NR < 0.1), the correspondent plasma values showed lactate 3.7 mmol/L and pyruvate 0.12 mmol/L (NR 0.03–0.09). She was discharged from the hospital at 2 weeks of age, slightly hypotonic and bottle‐fed. She thrived until 6 weeks of age. From then onwards she had diarrhea and difficulties gaining weight, lactate was persistently elevated (4–8 mmol/L) and transaminases borderline increased. A nasogastric tube was inserted at 4.5 months of age. Repeated echocardiography showed massive left ventricular hypertrophy (end‐diastolic septum thickness *Z*‐score 6 and left ventricular wall thickness at end‐diastole *Z*‐score 4) with left ventricular outlet obstruction but with preserved biventricular function. She was started on β‐blocker therapy and regained the ability to feed by bottle. HC increased from 50 (birth) to 97.5 centiles at 4.5 months of age. She had sun‐setting eyes‐sign and a full anterior fontanel. Ophthalmoscopy revealed no abnormalities. Cerebral MRI showed bilateral enlargement of subarachnoid spaces and a small, nonhemorrhagic subdural fluid collection on the left side (Figure S1d). No signal abnormalities or restricted diffusion were observed in brain parenchyma. Myelination was equivalent to 4‐months age. Both lateral ventricles and third ventricles were enlarged and the corpus callosum was slightly thinned, which taken together suggested a modest degree of brain atrophy (Figure S1e). These findings are though uncertain since myelination was slightly delayed and the corpus callosum might probably be thicker after complete myelination. Electroencephalography (EEG) was normal for age. Her cardiac condition/heart failure gradually deteriorated after 5 months, causing secondary feeding problems and lung congestion. She was palliated with morphine and died almost 7 months old.

Analysis of the oxidative phosphorylation (OXPHOS) enzyme activities in both patients was performed as previously described (Frazier & Thorburn, 2012; Rodenburg, 2011). A skeletal muscle biopsy and cultured skin fibroblasts of Subject 1 showed an isolated complex IV deficiency, whereas the activities of the other enzyme complexes were within the reference range (Table 1). The muscle histology and histochemistry were normal, no COX‐negative fibres were observed with the COX/SDH stain, which could be explained by the residual complex IV activity.

**Table 1:** Enzyme activities of OXPHOS complexes in skeletal muscle biopsy and fibroblasts of Subject 1 and skeletal muscle biopsy of Subject 2

	CI	CII	CIII	CIV	CV	CS (mU/mg)
Subject 1						
Skeletal muscle	506	440	326	**14**	–	91
Reference range (*n* = 35) (mU/UCS)	222–474	280–382	72–402	39–59	–	85–179
Fibroblasts	290	480	616	**51**	613	364
Reference range (*n* = 109) (mU/UCS)	163–599	335–888	570–1383	288–954	193–819	151–449
Subject 2						
Skeletal muscle	217	237	577	**124**	–	408
Reference range) (*n* = 9) (mU/UCS)	68–230	76–280	182–1421	228–1032	–	111–604

*Note*: All measurements are performed in duplicate and were only accepted when each of the duplicate values was within a 10% range of their average.

Activities are expressed as milliunits per unit citrate synthase (mU/U CS) or mU/mg protein for citrate synthase. Complex IV activity is clearly reduced in both patients in muscle and fibroblasts (indicated in bold). NB in skeletal muscle of Subject 1, complex III and complex IV are calculated as rate constants rather than initial rates (Frazier & Thorburn, 2012).

Abbreviation: OXPHOS, oxidative phosphorylation.

A skeletal muscle biopsy of Subject 2 showed an isolated complex IV deficiency (Table 1), and high‐resolution respirometry of the skeletal muscle analyzed with an Oxygraph‐2k (Oroboros Instruments) showed clearly reduced mitochondrial respiration through complex I–V with the substrates pyruvate + malate + glutamate + ADP and succinate (Table S1). To identify the genetic cause of the complex IV deficiency we performed WES and detected a homozygous variant in the gene encoding the complex IV assembly factor COX16 in both patients: Chr14(GRCh37): g.70793127G>A; NM_016468.6: c.244C>T p.(Arg82*). The variant has been submitted to LOVD (https://databases.lovd.nl/shared/genes/COX16). It creates a premature stop codon in exon 4. The variant is present with a very low allele frequency of 0.0034% in the gnomAD database with no homozygotes reported (https://gnomad.broadinstitute.org/). No other (potentially) pathogenic variants were detected in the nuclear genes encoding the complex IV subunits nor in one of the known assembly factors for complex IV. Furthermore, the complete mitochondrial DNA (mtDNA) of both patients was screened for mismatches and mtDNA rearrangements using long template polymerase chain reaction and the Ion Torrent PGM. This showed no pathogenic abnormalities in both patients. Segregation analysis revealed that the parents of both patients were heterozygous for the identified COX16 variant.

To analyze COX16 and RC complex protein expression, we performed immunoblot experiments. Blue native polyacrylamide gel electrophoresis (BN‐PAGE) with 2% wt/wt *n*‐dodecyl β‐d‐maltoside solubilized mitochondrial proteins from Subject 1 fibroblasts and subsequent Western blot analysis showed an absence of the fully assembled complex IV with normal expression levels of the other OXPHOS complexes (Figure S2a). In addition, sodium dodecyl sulfate‐PAGE (SDS‐PAGE)/Western blot showed undetectable levels of the COX16 protein and the COX2 subunit of complex IV, and reduced expression levels of the complex IV subunits COX1, COX4, and COX5As (Figure S2b). Similar observations were made by SDS‐PAGE/Western blot of the skeletal muscle biopsy of Patient S2 (Figure S2c).

**Figure 1:**
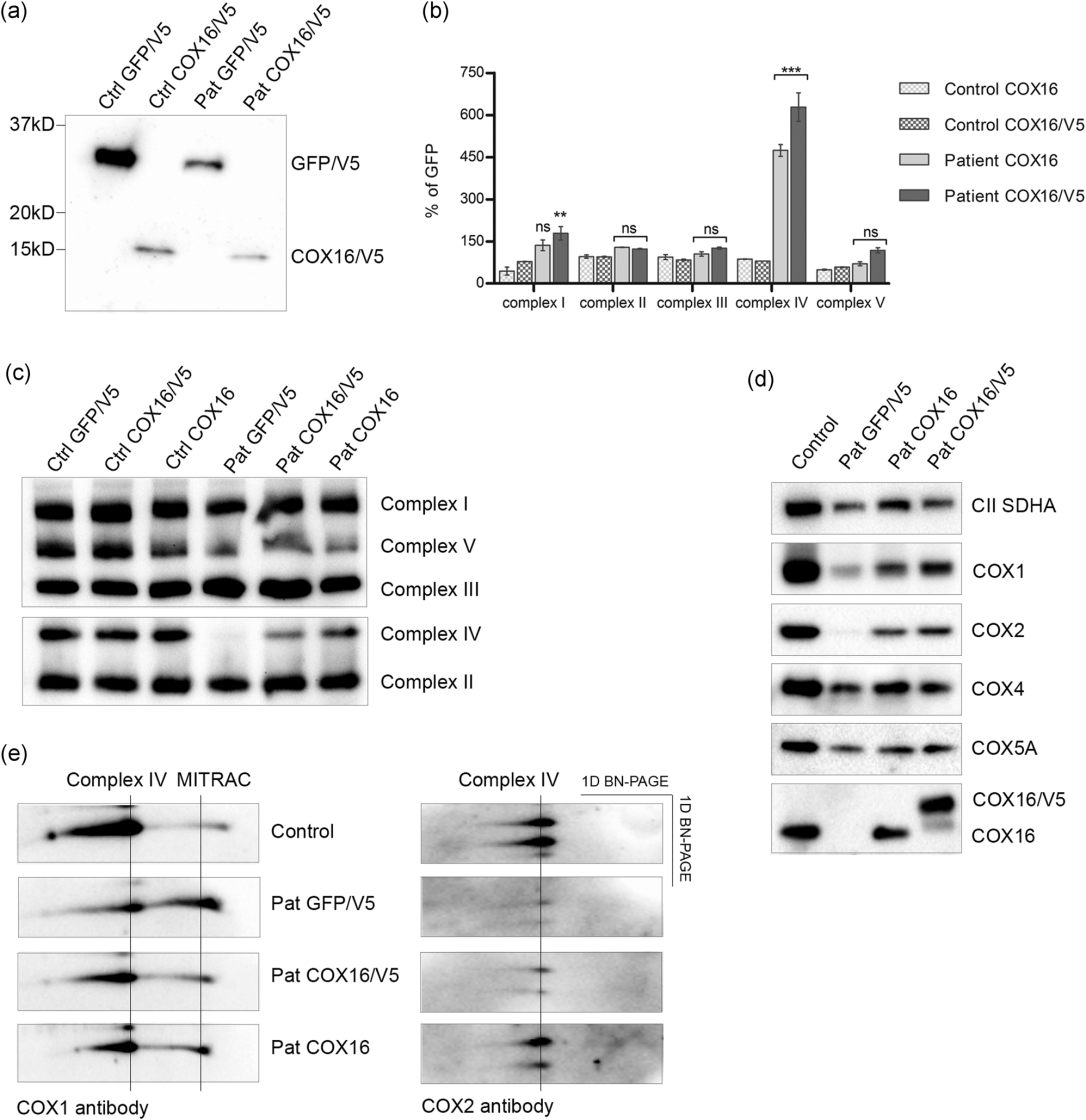
Lentiviral complementation of patient (Pat) fibroblasts with wild‐type *COX16* complementary DNA (cDNA) with (COX16/V5) or without V5 tag (COX16) rescues complex IV deficiency: (a) Sodium dodecyl sulfate–polyacrylamide gel electrophoresis (SDS‐PAGE)/Western blot of mitochondrial fractions of the transduced Patient (Pat) and control (Ctrl) cell lines shows an expression of the transgenic proteins GFP/V5 and COX16/V5 detected with the anti‐V5 antibody. (b) Graph of the relative activities of the oxidative phosphorylation (OXPHOS) enzymes in COX16‐transduced Pat and Ctrl cell lines expressed as a percentage of the activities of the GFP‐transduced in the same cell lines (%GFP). This shows a significant rescue of complex IV activity in the *COX16* cDNA‐transduced Pat cell lines (Pat COX16 and Pat COX16/V5) (***p < .001, two‐way analysis of variance [2way ANOVA]). Except for the minor change in complex I transduced with *COX16/V5* cDNA (**p < .01, two 2way ANOVA), there are no significant changes in the relative activities of the other OXPHOS enzymes (ns). The error bars indicate standard deviation. C) Blue native‐PAGE (BN‐PAGE)/western blot of mitochondrial fractions of the transduced Pat and Ctrl cell lines with wild‐type *COX16* cDNA shows a clear rescue of the assembly of complex IV in the Pat cell lines, whereas the *GFP* cDNA complemented Pat cell line has undetectable complex IV. Antibodies were used against NDUFA9 for complex I, UQCRC2 for complex III, ATP5A for complex V, COX4 for complex IV, SDHA for complex II. (d) SDS‐PAGE/Western blot of mitochondrial fractions of the transduced Pat and Ctrl cell lines shows a clear expression of both the COX16 and the transgenic COX16/V5 proteins. In addition, the expression levels of all the tested subunits, COX1, COX2, and to a lesser extend COX4 and COX5A, are increased in Pat cells expressing wild‐type *COX16* cDNA. Antibodies were used against COX16, COX1, COX2, COX4, COX5A; loading Ctrl CII SDHA subunit was detected with anti‐SDHA. (e) 2D‐BN‐PAGE/SDS‐PAGE of mitochondrial fractions (60 µg of protein) of wild‐type *COX16* cDNA and *GFP* cDNA‐transduced Pat cell lines are compared with a Ctrl cell line to analyze subassembly products. The Pat + GFP shows more COX1 in the mitochondrial translation regulation assembly intermediate of cytochrome c oxidase assembly intermediate than Ctrl cells; this reduces and shifts towards the holo‐complex after complementation with COX16/V5 and COX16. COX2 assembly intermediates are not visible and COX2 is barely visible in the holo‐complex in the Pat + GFP. After complementation with both *COX16* cDNAs, the COX2 could be detected in the holo‐complex. Antibodies were used against COX1 and COX2 subunits. The Western blot data in this figure was obtained in *N* = 1 independent experiment for (a) and (e), and *N* = 2 for (c) and (d). The data of the enzyme activities of (b) are obtained in *N* = 1 experiment with two duplicates. Duplicates are measured in two independent experiments and were only accepted when each of the duplicate values was within a 10% range of their average

Next, we performed lentiviral transduction to analyze whether the complex IV deficiency could be rescued by expressing wild‐type (wt) COX16. For this purpose, wt‐*COX16* complementary DNA (cDNA) either with or without a C‐terminal V5‐tag, or green fluorescent protein (GFP) as a control, were transduced in the patient fibroblasts of Subject 1 and healthy control fibroblasts. The expression of the transgenes was confirmed by SDS‐PAGE/Western blots with V5 and COX16 antibody (Figures 1a and 1d). BN‐PAGE/immunoblotting of the RC complexes showed a clear increase of fully assembled complex IV in patient fibroblasts complemented with wt‐*COX16* in comparison to the *GFP*‐transduced patient cells (Figure 1c). The expression levels of several complex IV subunits of the patient fibroblasts complemented with wt‐*COX16* and *GFP* were analyzed with SDS‐PAGE/Western blot analysis. The results showed increased levels of all the tested subunits, COX1, COX2, and to a lesser extent COX4 and COX5A only in cells complemented with wt‐*COX16* (Figure 1d). Activity measurements of the RC enzymes showed a complete rescue of complex IV activity in cells transduced with the wt‐*COX16* construct compared to the *GFP*‐transduced patient cell lines. Except for the slightly elevated complex I activity in the patient cell line transduced with *COX16/V5*, there are no significant changes in the relative activities of the other OXPHOS enzymes both in patient and control cell lines. Although complex I is not deficient in the patient cells, it might be affected by the normalization of complex IV after complementation, providing a more normal context for complex I, given the fact that subfractions of complex I and IV are normally present in the same supercomplexes. (Figure 1b). These results are in agreement with the notion that *COX16* is the causal disease gene in the patient. To investigate which part of the assembly of complex IV is affected by the COX16 deficiency we performed 2D‐BN‐PAGE/SDS‐PAGE immunoblotting, as previously described (Calvaruso et al., 2008). We observed an accumulation of COX1 in the mitochondrial translation regulation assembly intermediate of cytochrome c oxidase in the patient cell line transduced with *GFP*, whereas COX2 was barely visible in the holo‐complex (Figure 1e). The introduction of wt‐*COX16* resulted in a significant increase of the amount of COX1 in the holo‐complex, and COX2 could be detected in the fully assembled complex IV. Recent studies in which the expression of the *COX16* gene in HEK293 cells was eliminated using CRISPR/Cas9 showed similar results (Aich et al., 2018; Cerqua et al., 2018): a decrease of subunit COX2 and an accumulation of a COX1 containing subassembly product. However, in that model system the expression levels of the COX1, COX4, and COX5A subunits were similar to those in the control cells, and the complex IV activity appeared to be less severely reduced (50%–65% of the lowest reference value) than in the patients described in this report. This all demonstrates an important role for COX16 in the formation of the COX2 module.

Data from recent studies in human HEK293 cells suggested that COX16 plays a role in the assembly of the Cu_A_ center of the COX2 module (Cerqua et al., 2018; Signes & Fernandez‐Vizarra, 2018), however, its precise function is still unknown. Copper is integrated at two stages of complex IV assembly: into COX1 (Cu_B_ center) and into COX2 (Cu_A_ center). Patients with pathogenic genetic variants in genes encoding proteins involved in the copper delivery into the Cu_A_ center, such as *SCO1* (Leary et al., 2013), *SCO2* (Hallas et al., 2018; Jaksch et al., 2000; Papadopoulou et al., 1999), and *COA6* (Baertling et al., 2015; Ghosh et al., 2014), have been documented to present with infantile mitochondrial encephalomyopathy and cardiomyopathy, which is a similar clinical phenotype as the COX16 patients described here. Copper treatment of patient cell lines with isolated complex IV deficiency due to pathogenic variants in *SCO1, SCO2*, and *COA6* genes, all part of the copper delivery route, has previously been shown to either partially or fully restore the complex IV activity (Baertling et al., 2015; Casarin et al., 2012; Jaksch et al., 2001; Salviati et al., 2002). In the study of Cerqua et al. (2018) similar effects were observed in *COX16*‐knock out HEK293 cells. To investigate whether copper‐treatment might have a positive effect on complex IV in our patient cells, we treated the fibroblast cell line of Subject 1 and a control cell line for 72 h with concentrations between 0 and 100 µM of CuCl_2_ and performed SDS‐PAGE and BN‐PAGE with subsequent immunoblotting. No positive effect of the copper treatment on the levels of COX subunits or the amount of fully assembled complex IV was observed. In addition, the complex IV enzyme activity showed no recovery at all after the CuCl_2_ treatment of the patient cell line (Figure S3a–c).

In the report of Aich et al. (2018) it is stated that instead of delivering copper to the COX2 module, COX16 could be involved in the recruitment of the metallochaperone SCO1 to the COX2 subunit. Our results from the copper treatment of the patient cells would be in agreement with this hypothesis: our observation that supplementing copper does not increase complex IV activity can be explained by the absence of COX16 to recruit SCO1 to the COX2 module. The fact that COX16 lacks conserved cysteine and histidine residues that are usually present in classical copper‐binding motifs (Koch et al., 1997) reinforce this hypothesis. In addition, Aich et al. (2018) suggested that COX16 could play a role in merging the COX1 and COX2 assembly lines. In the COX16‐deficient cell line of Subject 1, we observed reduced levels of the COX1 subunit, in addition to almost nondetectable levels of the COX2 subunit. These results are in agreement with the findings of Aich et al. (2018).

In conclusion, this is the first report of two unrelated patients with a pathogenic variant in the *COX16* gene responsible for the complex IV deficiency leading to neonatal hypertrophic cardiomyopathy, encephalopathy, and severe lactic acidosis with fatal outcome. Our data demonstrate that COX16 deficiency is a cause of mitochondrial disease.

## CONFLICT OF INTERESTS

The authors declare that there are no conflict of interests.

## Supporting information

Supporting information.

Supporting information.

## Data Availability

The data that support the findings of this study are available on request due to privacy/ethical restrictions.
