# Supplementary material for: A novel variant in COX16 causes cytochrome c oxidase deficiency, severe fatal neonatal lactic acidosis, encephalopathy, cardiomyopathy, and liver dysfunction
Source: Hum Mutat. 2020 Nov 30;42(2):135–41. doi: 10.1002/humu.24137 (PMC7898715; doi:10.1002/humu.24137)
Supplement: Supplementary file 1 — Supporting information. [file HUMU-42-135-s001.pdf]

SUPLEMENTARY DATA

MATERIALS & METHODS

Sample collection

For subject 1, blood for DNA was collected on day 2, and muscle and skin were collected on day 3 of life. For subject 2 blood was collected in the first month of life, a skeletal muscle biopsy was taken at 4,5 month of age.

Whole exome sequencing and Sanger sequencing

Whole exome sequencing and data analysis of subject 1 were performed in the Netherlands, Nijmegen as described before(Neveling et al., 2013; Wortmann, Koolen, Smeitink, van den Heuvel, & Rodenburg, 2015). In short, exome enrichment was performed using the SureSelect Human All Exon 50Mb Kit V5 (Agilent). Sequencing was done on an Illumina HiSeq sequencing platform with median coverage of 100x. Analysis of the sequencing data followed the practice from Genome Analysis Tool Kit (GATK) for exome sequencing. Read alignment was performed to the human reference genome (GrCH37/hg19). Variant annotation was performed using a custom designed in-house annotation, where after these were filtered in several steps to exclude intronic (except for splice sites), synonymous changes and common variants. This was done by comparison with dbSNPv132 (https://www.ncbi.nlm.nih.gov/snp), Exome Variant Server (NHLBI ESP: http://evs.gs.washington.edu/EVS), Exome Aggregation Consortium (ExAC: http://exac.broadinstitute.org), Genome Aggregation Database (gnomAD: https://gnomad.broadinstitute.org), or in our in-house genome database.

Segregation analysis of the parents of subject 1 was performed by Sanger sequencing.

For subject 2 and her parents, the whole exome sequencing and data analysis was performed in Norwegian, Oslo as described in McKenna et al., 2010 (McKenna et al., 2010), annotation was done by Annovar (http://wannovar.wglab.org) (Wang, Li, & Hakonarson, 2010). Downstream filtering and analysis was done with Filtus (Vigeland, Gjotterud, & Selmer, 2016) on the variants within coding

regions and intron/exon boundaries. A Trio-based inheritance filtering was used focusing on clinically-relevant de-novo, recessive or X-linked variants. Synonymous changes and common variants (in gnomAd and/or in our in-house database) were excluded.

The complete mtDNA of both patients was screened for mismatches and mtDNA rearrangements using Long Template PCR and the Ion Torrent PGM in the Netherlands, Nijmegen.

Cell culturing, mitochondrial isolation, enzymatic analysis & high resolution respirometry analysis

Patient and control fibroblasts were cultured in M199 medium (Gibco) supplemented with 10% v/v fetal calf serum (FCS) and 1% v/v penicillin/streptomycin (Gibco) at 37 ^{o}C with 5% CO_2. Mitochondrial fractions were isolated for the analysis of the RC enzyme activities, SDS-PAGE, BN-PAGE and 2D BN-PAGE/SDS-PAGE. In short, fibroblasts were disrupted by a Potter-Elvejhem homogenizer in in a hypotonic 10mM Tris buffer, sucrose (250mM) was added to make the samples isotonic and the mitochondrial fraction was derived by differential centrifugation.(Janssen et al., 2007)

The activities of the OXPHOS complexes, citrate synthase (CS) and total protein in the mitochondrial fractions of the fibroblasts of subject 1 and in the 600g supernatant of the frozen skeletal muscle of subject 2 were measured spectrophotometrically as previously described (Rodenburg, 2011), using a Konelab 20XT auto-analyser. Activities of OXPHOS enzymes, CS and total protein in the skeletal muscle of subject 1 were measured spectrophotometrically in post-600g supernatant as previously described (Frazier & Thorburn, 2012). OXPHOS enzyme activities were normalized on the activity of citrate synthase.

High-resolution respirometry was performed on isolated mitochondria from the Musculus Vastus Lateralis of a fresh muscle of subject 2 with the Oxygraph-2k (Oroboros Instruments). Muscle tissue was homogenized in ice cold SETH (0,25 M Sucrose 2,0 mM EDTA 10 mM Tris, 5 x 10^4 U/L Heparin) immediately after biopsy, mitochondria were isolated by differential centrifugation in 2

steps of 600g followed by 10.000g. The mitochondrial pellets were washed once and after resuspension in ice cold SETH transferred to the preequilibrated oxygraphy chamber with mitochondrial respiratory medium Mir05(Gnaiger, 2014). Analysis of O_2 consumption was performed by using the substrate-uncoupler-inhibitor titration (SUIT) protocol at 37 °C with constant stirring (Gnaiger, 2014). Mitochondrial respiration was calculated using Datlab7 software and expressed as nmol $O_2 .s^{-1}.g$ protein^{-1}.

Lentiviral complementation of patient fibroblasts

Wild type *COX16* cDNA with and without a stopcodon, or *GFP* cDNA were cloned into a pDONR201 vector and recombined with pLenti6.2/V5-DEST Gateway Vector using the Gateway LR Clonase II enzyme mix (Invitrogen) as previously described (Nouws et al., 2010)

Lentiviral particles were produced by transfecting HEK293T cells according to the manufacturer's protocol (Invitrogen). Patient and control cell lines were transduced and selection for stable *COX16-V5*, untagged *COX16* or *GFP-V5* transduced cells was achieved by growing the cells on culture medium with blasticidin (2.5 μg/ml) for 14 days. Blasticidin resistant cells were used for functional analysis.

Copper treatment

Patient and control fibroblasts were treated with different concentrations of copper ($CuCl_2$).

A stock solution of 500 µM $CuCl_2$ was prepared by dissolving $CuCl_2$ (Sigma) in M199 medium. Final concentrations of 25, 50 and 100µM $CuCl_2$ were obtained by further dilution of the stock solution in M199 medium. Cell lines were incubated for 72 hr and harvested for enzymatic analysis, SDS-PAGE and BN-PAGE/western blotting.

SDS-PAGE, 2D-BN-PAGE/SDS-PAGE and immunoblotting

SDS-PAGE was performed using 12% precast Trupage gels (Sigma). For BN-PAGE 6-16% precast NativePAGE Bis-Tris gels (Invitrogen) were used. Mitochondrial or 600g supernatant fractions were either processed for SDS-PAGE by adding SDS-PAGE sample buffer or solubilized with 2% w/w *n*-dodecyl β-D-maltoside for BN-PAGE

For two-dimensional BN-PAGE/SDS-PAGE the native mitochondrial complexes (60 µg of protein) were separated on a 4-12% BN gradient gel followed by the second dimension on a denaturing SDS-PAGE using a 12 % SDS-PAGE gel as described previously (Calvaruso, Smeitink, & Nijtmans, 2008). Subsequent western blotting was done on a PVDF membrane and immunodetection was performed with the following antibodies: COX1 (ab14075; Abcam), COX2 (MS405; Mitosciences), COX4 (MS407; Mitosciences), COX5A (MS409; Mitosciences), CI -NDUFB11 (ab183716, Abcam), CI-NDUFA9 (ab14713, Abcam), CII-SDHA (Ab14715; Abcam), CIII- UQCR Core2 (ab14745; Abcam), CIII-UQCR Core1(MS303; Mitosciences) CV-5A (Ab14748, Abcam), V5 (R960 25; Invitrogen), COX16 (19425-1-AP; Proteintech). Secondary antibodies: goat anti-mouse (P0047; DAKO) and goat anti-rabbit (A00160, Genscript). The chemiluminescence signal was visualized using the enhanced chemiluminescence kit (ECL, Thermo Fischer Scientific) and the Chemidoc XRS+ system (Biorad).

WEB RESOURCES

dbSNPv132: https://www.ncbi.nlm.nih.gov/snp

Exome Variant Server, NHLBI ESP: http://evs.gs.washington.edu/EVS/

Exome Aggregation Consortium, ExAC: http://exac.broadinstitute.org/

Genome Aggregation Database, gnomAD: https://gnomad.broadinstitute.org/,

Annovar: http://wannovar.wglab.org/

REFERENCES

Calvaruso, M. A., Smeitink, J., & Nijtmans, L. (2008). Electrophoresis techniques to investigate defects in oxidative phosphorylation. *Methods, 46*(4), 281-287. doi:10.1016/j.ymeth.2008.09.023

Frazier, A. E., & Thorburn, D. R. (2012). Biochemical analyses of the electron transport chain complexes by spectrophotometry. *Methods Mol Biol, 837*, 49-62. doi:10.1007/978-1-61779-504-6_4

Gnaiger, E. (2014). *Mitochondrial pathways and respiratory control. An introduction to OXPHOS analysis. Mitochondr Physiol Network 19.12* (Vol. 80): OROBOROS MiPNet Publications, Innsbruck.

Janssen, A. J., Trijbels, F. J., Sengers, R. C., Smeitink, J. A., van den Heuvel, L. P., Wintjes, L. T., . . . Rodenburg, R. J. (2007). Spectrophotometric assay for complex I of the respiratory chain in tissue samples and cultured fibroblasts. *Clin Chem, 53*(4), 729-734. doi:10.1373/clinchem.2006.078873

McKenna, A., Hanna, M., Banks, E., Sivachenko, A., Cibulskis, K., Kernytsky, A., . . . DePristo, M. A. (2010). The Genome Analysis Toolkit: a MapReduce framework for analyzing next-generation DNA sequencing data. *Genome Res, 20*(9), 1297-1303. doi:10.1101/gr.107524.110

Neveling, K., Feenstra, I., Gilissen, C., Hoefsloot, L. H., Kamsteeg, E. J., Mensenkamp, A. R., . . . Nelen, M. R. (2013). A post-hoc comparison of the utility of sanger sequencing and exome sequencing for the diagnosis of heterogeneous diseases. *Hum Mutat, 34*(12), 1721-1726. doi:10.1002/humu.22450

Nouws, J., Nijtmans, L., Houten, S. M., van den Brand, M., Huynen, M., Venselaar, H., . . . Vogel, R. O. (2010). Acyl-CoA dehydrogenase 9 is required for the biogenesis of oxidative phosphorylation complex I. *Cell Metab, 12*(3), 283-294. doi:10.1016/j.cmet.2010.08.002

Rodenburg, R. J. (2011). Biochemical diagnosis of mitochondrial disorders. *J Inherit Metab Dis, 34*(2), 283-292. doi:10.1007/s10545-010-9081-y

Vigeland, M. D., Gjotterud, K. S., & Selmer, K. K. (2016). FILTUS: a desktop GUI for fast and efficient detection of disease-causing variants, including a novel autozygosity detector. *Bioinformatics, 32*(10), 1592-1594. doi:10.1093/bioinformatics/btw046

Wang, K., Li, M., & Hakonarson, H. (2010). ANNOVAR: functional annotation of genetic variants from high-throughput sequencing data. *Nucleic Acids Res, 38*(16), e164. doi:10.1093/nar/gkq603

Wortmann, S. B., Koolen, D. A., Smeitink, J. A., van den Heuvel, L., & Rodenburg, R. J. (2015). Whole exome sequencing of suspected mitochondrial patients in clinical practice. *J Inherit Metab Dis, 38*(3), 437-443. doi:10.1007/s10545-015-9823-y
