# Supplementary material for: A novel variant in COX16 causes cytochrome c oxidase deficiency, severe fatal neonatal lactic acidosis, encephalopathy, cardiomyopathy, and liver dysfunction
Source: Hum Mutat. 2020 Nov 30;42(2):135–41. doi: 10.1002/humu.24137 (PMC7898715; doi:10.1002/humu.24137)
Supplement: Supplementary file 2 — Supporting information. [file HUMU-42-135-s002.pdf]

Supplementary Figures, and Tables with legends

Figure S1

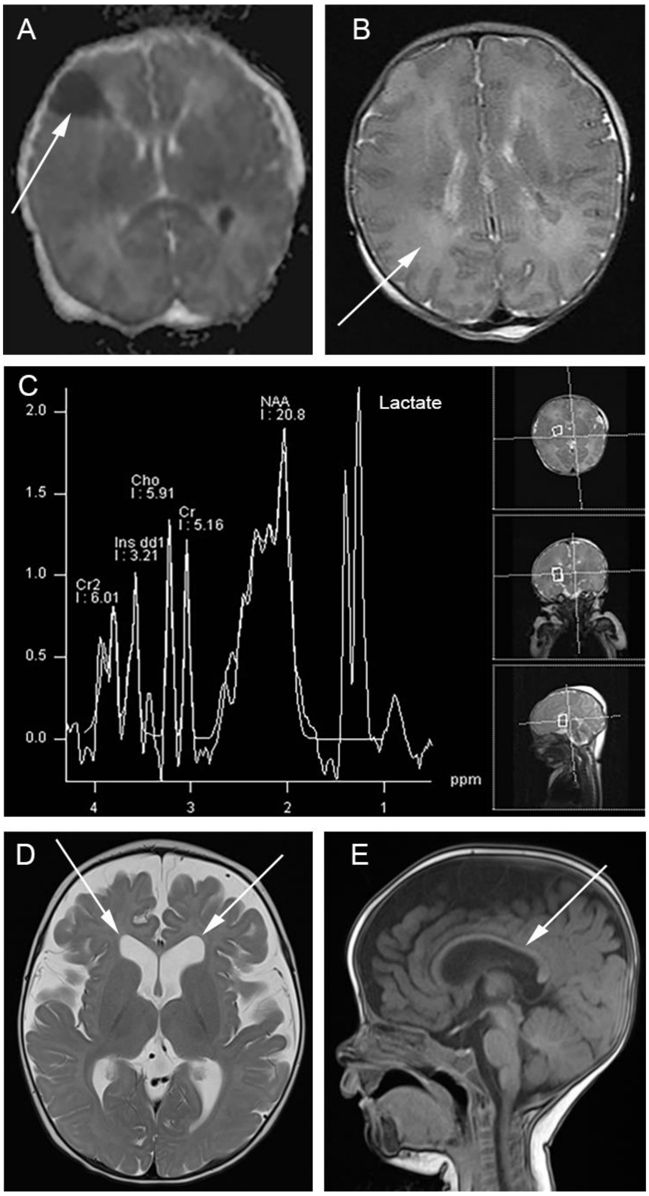

Figure S1. MR and MS images of both patients.

A) Axial Diffusion weighted image (DWI) and B) Axial T2-weighted MR Image of subject 1 show intense cerebral diffusion restriction with multiple areas of restricted diffusion in bilateral posterior occipital lobes (arrow on A), right frontal cortical/deep white matter region (arrow on B), bilateral posterior limbs of the internal capsule, posterior left thalamic pulvinar and bilateral posterior

centrum semiovale lateral to the posterior horns of lateral ventricles. There is also loss of grey-white differentiation. These findings are suggestive of multiple infarcts with generalised cerebral edema.

C) The MR spectroscopy of subject 1 demonstrates an increased lactate peak.

D) Axial and E) Sagittal T1-weighted MR images of subject 2 show bilateral enlargement of subarachnoid spaces. No signal changes in brain parenchyma were observed. Both lateral ventricles and third ventricle are enlarged (arrows D) and corpus callosum is slightly thinned (arrow E), suggesting a modest degree of brain atrophy, although myelination is slightly delayed and corpus callosum might be thicker after complete myelination.

Figure S2

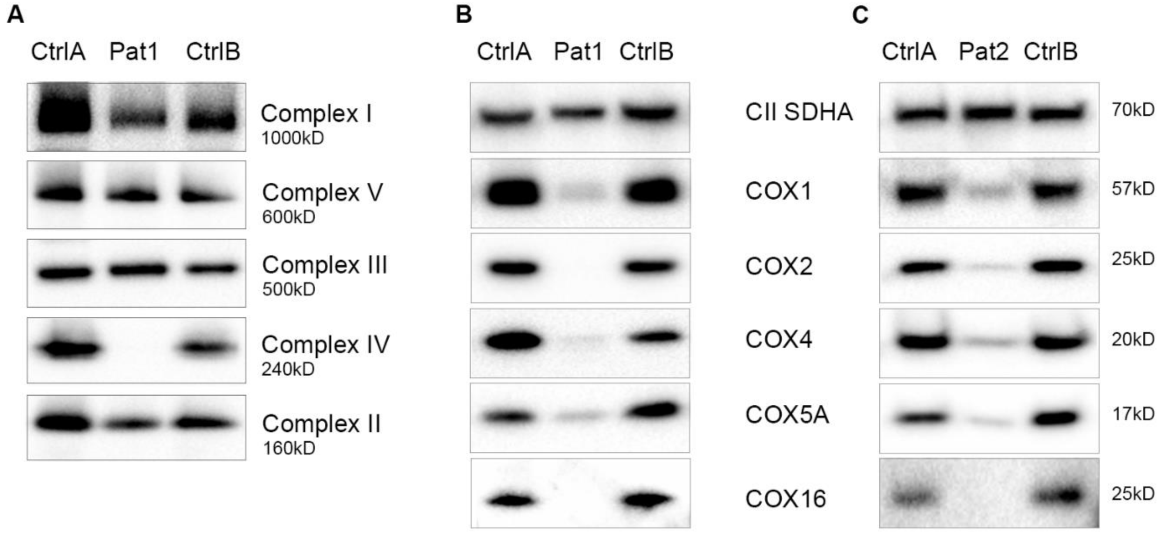

Figure S2. Western blots of patient samples show reduced levels of complex IV, complex IV subunits and COX16 protein.

A) BN-PAGE/western blot of mitochondrial fractions of subject 1 (Pat1) fibroblasts and two control(CTRL A & B) fibroblast cell lines. Complex IV (anti-COX4) is hardly detectable whereas the levels of the other OXPHOS complexes, complex I (anti-NDUFB11), complex V (anti-ATP5A), CIII (anti-UQCRC2) and complex II (anti-SDHA) are comparable to the controls. B) SDS-PAGE/western blot of

mitochondrial fractions of fibroblasts of subject 1 (Pat1) and 2 controls (CTRL A & B) shows undetectable levels of COX16 and subunit COX2, and reduced levels of the subunits COX1, COX4 and COX5A in the patient cell line; CII subunit SDHA serves as a loading control.

C) SDS-PAGE/western blot of 600g supernatant of skeletal muscle of subject 2 (Pat2) and of 2 healthy controls (CTRL A & B)shows an undetectable level of COX16, and reduced levels of the subunits COX1, COX2, COX4 and COX5A.

On both SDS-PAGE/western blots antibodies were used against COX16, COX1, COX2, COX4, COX5A; loading control CII SDHA subunit was detected with anti-SDHA. Results were obtained in N=2 independent experiments for A and B, and N=1 for C

Figure S3

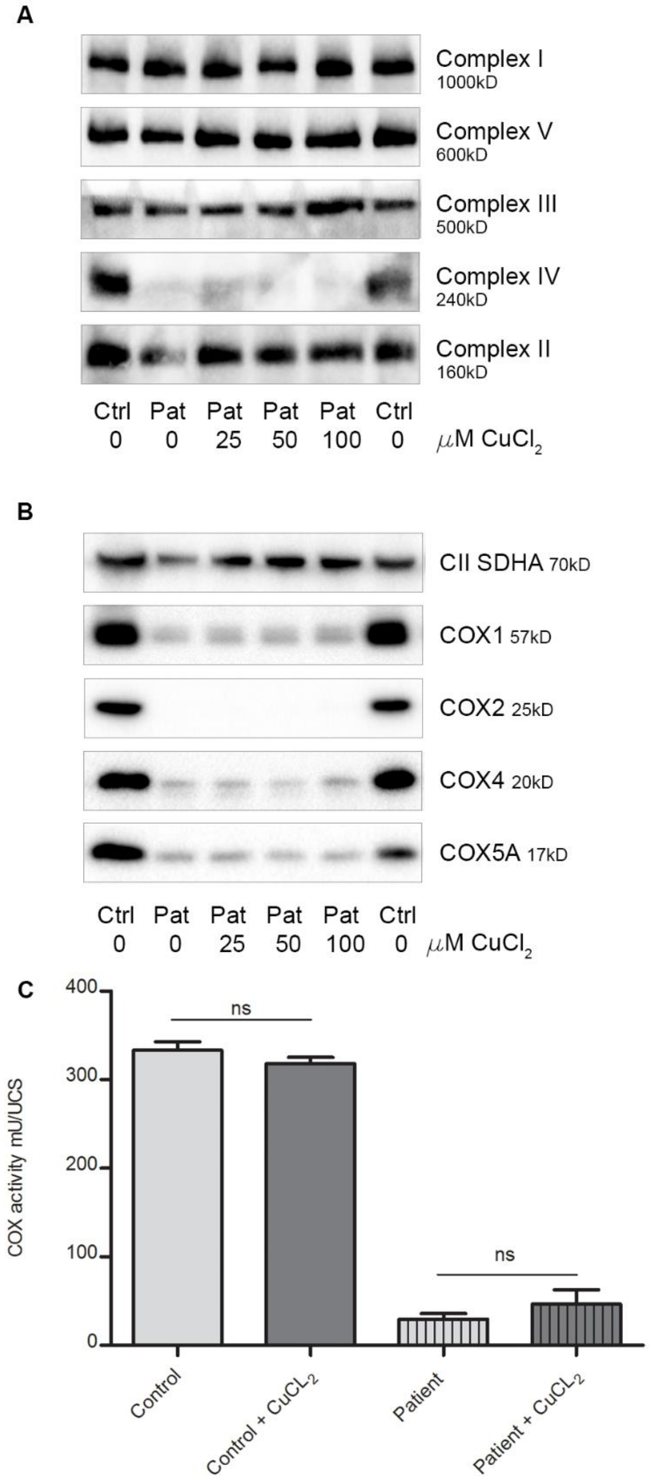

Figure S3. Effect of Copper treatment on patient subject 1 and control fibroblasts.

A) BN-PAGE/western blot of mitochondrial fractions of patient (Pat) and control (Ctrl) fibroblast cell lines after treatment with Copper ($CuCl_2$) in concentrations of respectively 25, 50 and 100 µM shows no effect on the level of complex IV (anti-COX4). Levels of the other OXPHOS complexes, complex I

(anti-NDUFB11), complex V (anti-ATP5A), complex III (anti-UQCRC2) and complex II (anti-SDHA) remain unchanged. Results were obtained in N=2 experiments

B) SDS-PAGE/western blot of mitochondrial fractions of patient (Pat) and control (Ctrl) fibroblasts shows no effect on the expression of the subunits COX1, COX2, COX4 and COX5A after treatment with increasing concentrations of $CuCl_2$; the SDHA subunit of complex II serves as a loading control. Antibodies were used against COX1, COX2, COX4, COX5A; and-SDHA. Results were obtained in N=2 experiments

C) Complex IV activities of mitochondrial fractions of patient and control fibroblasts after copper treatment (100 µM $CuCl_2$). The patient cell line shows no improvement of the complex IV (COX) activity. All measurements are performed in duplicate and were only accepted when each of the duplicate values was within a 10% range of their average. The error bars indicate standard deviation, P = ns (not significant) in an unpaired *t*-test.

Table S1

**Subject 2**	Pyruvate + Malate + Glutamate +ADP	Succinate
**Skeletal Muscle**	1158	874
**Reference range (nmol .s^{-1}.g $prot^{-1}$)**	3470 -10500	2810 -11500

Table S1: High-resolution respirometry analysis of the skeletal muscle of subject 2 reveals a clearly reduced mitochondrial respiration (nmol O_2 .s^{-1}.g $protein^{-1}$) through complex I-V with the substrates pyruvate+malate+glutamate+ADP and succinate. The assay is performed in one measurement with two individual samples, the reference range was determined in n=26 healthy volunteers (not age matched).
